# Health Risks and Potential Sources of Contamination of Groundwater Used by Public Schools in Vhuronga 1, Limpopo Province, South Africa

**DOI:** 10.3390/ijerph17186912

**Published:** 2020-09-22

**Authors:** John Ogony Odiyo, Mashudu Maxwell Mathoni, Rachel Makungo

**Affiliations:** Department of Hydrology and Water Resources, University of Venda, Thohoyandou 0950, South Africa; mashmathoni@gmail.com (M.M.M.); rachel.makungo@univen.ac.za (R.M.)

**Keywords:** agricultural activities, contamination, groundwater quality, health risks, pit latrines, school children

## Abstract

Public schools in most rural areas of South Africa depend on untreated groundwater due to unreliable water supply by the municipalities. This has the potential to cause water-related health problems to school children. Temperature, pH, and electrical conductivity (EC) were measured in situ. Chemical (fluoride, chloride, sulphate, phosphate, nitrate, magnesium, calcium, sodium, and potassium) and microbial (*Escherichia coliform (E. coli)*, *Salmonella typhimurium*, and *Shigella flexneri)* water quality parameters were analysed in groundwater samples from 10 public schools in Vhuronga 1 to determine suitability for use. Quantitative microbial risk assessment was carried out to determine risks of infection and illness due to consumption of groundwater. Correlation analysis was used to identify potential sources of contamination. All physical and most chemical water quality parameters were within guidelines for domestic water use. A high proportion of schools had high levels of microbial organisms. Risks of infection per day were relatively low for all schools. The annual risks of infection due to *E. coli* and *Shigella flexneri* for most schools was high, with maximum values of 89.11 and 83.75%, respectively. Maximum risks of illness per year were 31.19, 30.37, and 29.31% for *E. coli*, *Salmonella typhimurium*, and *Shigella flexneri*, respectively. Correlation analysis indicated potential contamination of groundwater by agricultural activities, domestic waste, and faecal contamination from pit latrines. Preventive and mitigation measures to minimise such risks, including locating boreholes at safe distances from pit latrines, prevention/minimisation of pollution of groundwater from agricultural activities, and point-of-use treatment of groundwater by the schools are therefore essential.

## 1. Introduction

Most public schools in South Africa are dependent on groundwater due to lack of and/or inadequate municipal water supply. Studies such as [1,2,3,4,5,6,7] have found that groundwater used by school children, mostly in rural areas of developing countries, is of poor quality and has negative health impacts. De Vera [1] reported that the bacterial count and total coliform in groundwater from deep wells in four selected public elementary schools in the Philippines did not meet the standards, and was required to be treated before drinking. Drinking water at schools in the Santarém region, Amazon, Brazil was acidic, had high nitrate and aluminium concentrations as well as presence of total coliform and *Escherichia coliform* (*E. coli*), and hence had negative health impacts on school children [2]. High concentrations of calcium, potassium, sodium, sulphates, chloride, and nitrate were recorded in groundwater in some of the schools in Lower Shire Valley, Malawi [3].

Groundwater used by school children in rural areas in the Giyani region, South Africa had poor microbiological quality, high diversity of pathogenic bacterial organisms (*Salmonella*, *Shigella*, *Vibrio species*, *Campylobacteria*, and *E. coli*), and high levels of antibiotic resistance by the isolated organisms [4]. Samie et al. [5] also indicated that groundwater from the schools studied by Samie et al. [4] was not suitable for human consumption based on hardness and high nitrate concentrations, and posed a serious threat to the health of school children. Nitrate concentration in a water sample from the borehole used by the primary school in the Thaba Nchu area in South Africa exceeded the standard by almost tenfold, and was linked to mining-related activities within the vicinity [6]. Fifty percent of students from a primary school in Siloam Village, South Africa had mottled teeth due to high fluoride in groundwater [7].

Nearly 1.7 billion cases of childhood diarrhoeal disease which results to deaths of around 525,000 children every year are reported globally [8]. Children are more susceptible to diseases than adults and therefore need a healthy school environment [9], including safe drinking water. It is therefore crucial to assess and monitor the quality of groundwater used by school children and identify potential health risks, as this will aid in identifying intervention strategies and preventive measures. Poor health of school children has an impact on learning if they become sick most of the time. Public schools in Vhuronga 1 rely on pit latrines for sanitation. This has the potential of faecally contaminating groundwater sources.

Diarrhoea is the most common disease in school children that is associated with water contaminated by pathogens through the faecal–oral route [9]. Studies such as [10,11,12,13] have indicated the prevalence of diarrhoea, and in certain instances, cholera and dysentery associated with contamination of borehole water in rural areas of South Africa. This makes children studying in rural public schools more susceptible to these diseases. Taonameso et al. [14] linked sporadic outbreaks of diarrhoea in children to contamination of groundwater in the Vhembe District. Students from a primary school in Siloam Village experienced stomach cramps and a running stomach after drinking water from a borehole which was within the vicinity of three pit latrines, and had high levels of total coliform [15]. School children spend at least 6–8 h at school, and in dry areas such as Vhuronga 1, they are likely to consume large quantities of water. In addition, groundwater is also used to prepare food from government feeding schemes. This makes them vulnerable to diseases if the groundwater is contaminated. This study assessed the suitability and health risks of groundwater used by public schools in the Vhuronga 1 Circuit, and determined the relationship between water quality parameters and potential sources of contamination. Health risk assessment is crucial in identification of risk of infection and illness of students due to consumption of groundwater. Correlation analysis aided in understanding the relationship between water quality parameters and identifying the potential sources of pollution. This was essential to provide evidence or information that can be used in devising strategies to minimise potential contamination and health risks. Assessing groundwater quality, health risk assessment, and developing strategies to protect aquifers from contamination are necessary aspects of water resource planning. The aspect on risks of infection and illness in an area where all this is unknown is crucial for authorities and creation of awareness on protection of groundwater resources.

## 2. Materials and Methods

**Figure 1:**
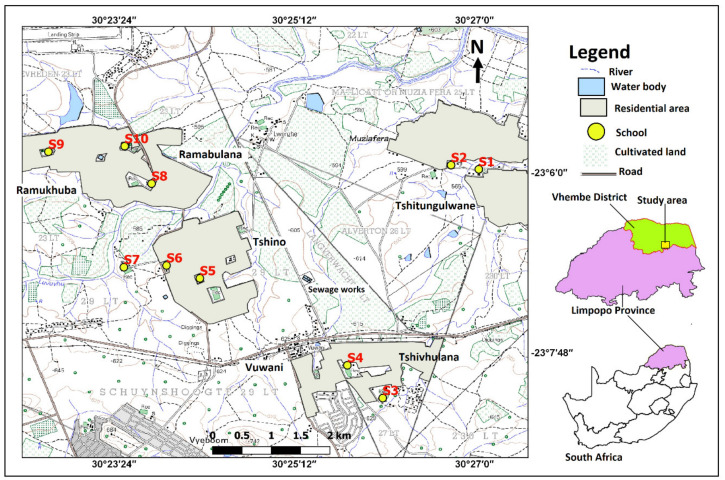
Location of the study area.

The Vhuronga 1 Circuit is located in the Vhembe District Municipality in Limpopo Province, South Africa (Figure 1). The area covers schools within the Tshitungulwane, Tshivhulana, Ramukhuba, Vuwani, and Tshino Villages. The schools are referred to as Schools 1–10 (S1−S10). The study area was under the Makhado Municipality water supply area. The surface water supply from the municipality is inadequate and unreliable. Due to this, most residents and the selected schools rely on groundwater from boreholes. Most of the study area has no established infrastructure for sewerage systems and residents rely on pit latrines for sanitation, though there are Vuwani sewage works which cater for the rural town of Vuwani. The main land use activities in the study area are residential settlements, agriculture, and natural forest (Figure 1). The areas covered by residential settlements, cultivated lands, and natural forest are 16.97, 19.90, and 4.43 km^2^, which respectively constitute 41, 48, and 11% of the total study area (41.30 km^2^). There are school gardens in all schools. S1 and S9 are the only schools where boreholes are located far away from the pit latrines, while boreholes in the rest of the schools are at distances of approximately 4 to 7 m from pit latrines.

Groundwater samples were collected once a month from 10 public schools within the Vhuronga Circuit 1 area from August to October 2018 (3 months). The samples were collected from storage tanks within the schools in sterile 1L plastic containers. In all the schools, groundwater is pumped directly from the sealed boreholes into the storage tanks. All samples were kept at 4–10 °C during transport to the laboratory and were analysed in triplicate for quality assurance purposes, and mean values were recorded. Samples were analysed for microbial parameters within 6 h, while those for chemical analysis were analysed within 24 h. Temperature, pH, and electrical conductivity (EC) were measured in situ using a Cyberscan 500 benchtop meter, while turbidity was measured using a Eutech TN 100 turbidity meter. Fluoride, chloride, sulphate, phosphate, and nitrate were analysed using 850 IC professional Ion Chromatography with a 25 injection loop, Ion Pac AG144 × 50mm guard column, and Ion Pac AS144 × 250mm analytical columns with the conductivity detector. The equipment was calibrated using 1, 5, 10, and 20 mg/L standards prepared from stock solutions following the manufacturer’s instructions. Magnesium, calcium, sodium, and potassium were analysed using the Varian 220 Atomic Absorption Spectrophotometer (AAS) attached to the GTA 110 electrothermal atomiser. A sterile syringe filter of 0.20 µm was used to filter the water samples. The instrument was calibrated using the prepared standard solutions for magnesium, calcium, sodium, and potassium. The instrument was then blanked using the prepared blank solutions before each parameter was analysed. The analysed physical and chemical water quality parameters were compared to the Department of Water Affairs and Forestry (DWAF) [16] guidelines for domestic water use to determine the suitability of groundwater for use.

Microbial analysis of target pathogens was conducted using the membrane filtration method. Water samples (100 mL) were passed through millipore filter membranes (pore size 0. 45 μm, 47 mm diameter). The filters were placed in petri dishes with M-Endo LES agar manufactured by Merck (Pretoria, South Africa) for presumptive enumeration of *E. coli*. M-Endo LES agar was prepared by suspending 51 g of its medium in 1 L of de-ionised water. Its mixture was heated with intermittent agitation until dissolution was complete, and it was dispensed into sterile petri dishes for further use. The petri dishes were placed in an incubator at 35 ± 2 °C for 24 h. The formation of a pink metallic sheen indicated the presence of *E. coli.*

For presumptive enumeration of *Salmonella typhimurium* and *Shigella flexneri*, millipore filter membranes were placed in petri dishes with bright red to reddish-orange Xylose Lysine Deoxycholate (XLD) agar manufactured by Acumedia (Pretoria, South Africa). The agar was prepared by suspending 55 g of its medium in 1 L of de-ionised water, which was heated with frequent agitation and boiled for one minute to completely dissolve the medium. It was immediately cooled in a water bath at 50 °C and poured into sterile petri dishes. The petri dishes were placed in an incubator at 37 °C for 24 h. *Shigella flexneri* was identified by characteristic red to pink colonies, while *Salmonella typhimurium* was identified by red colonies with a black center. For quality control, de-ionised water was used to prepare the agars and as a control test for the presence of *E. coil, Salmonella typhimirium*, and *Shigella flexneri*. All measurements were recorded as colony-forming units (CFU) per 100 mL. Microbial parameters were compared to WHO [17] and South African National Standards (SANS) [18] guidelines for domestic water use to determine the suitability of groundwater for use.

Risks of infection and illness of school children due to consumption of groundwater was assessed using a quantitative microbial risk assessment (QMRA). The QMRA procedure involved hazard identification, dose-response assessment, exposure assessment, and risk characterisation. Hazard identification involved identification of the pathogens and the nature of adverse health effects. In the dose-response assessment, the *E. coli* dose was multiplied by 0.08, since the literature has indicated that only 8% of *E. coli* is pathogenic [19]. The dose-response assessment which involved establishing the relationship between the dose of the pathogens and the probability of illness was based on the beta-Poisson model (Equation (1)). The dose-response relationships (Table 1) were obtained from the U.S. Department of Agriculture/Food Safety and Inspection Service and U.S. Environmental Protection Agency [20], which provides an overview of dose-response relationships for a range of waterborne pathogens from the existing literature.

(1) $$P_{\inf}\;=\;1\;-\;{\left(1\;+\;\frac{D}{\beta }\right)}^{-a},$$

where *P*_inf_ is the probability of infection per day, *D* is the average dose ingested, and *α* and *β* are the dose-response parameters. The average dose ingested was calculated by multiplying the consumed volume of water per day by the recorded average value of the enumerated pathogen for the three sampling periods. The consumed volume of water of 1 liter per day per child was adopted, following Ahmed [21].

For the exposure assessment, it was considered that school children were exposed to pathogens through consumption of groundwater at school. The number of school days for public schools in South Africa for the year 2018 was 203. The risks of annual infection and illness were calculated using Equations (2) and (3), respectively.

(2) $$P_{\inf_{annual}}\;=\;1\;-\;{\left(1\;-\;P_{\inf}\right)}^{n}$$

(3) $$P_{\mathrm{ill}}\;=\;P_{\inf_{annual}}\;\times \;P_{ill/in}$$

$P_{\inf_{annual}}$ is the probability of annual infection, n is the number of days of exposure in a year, *P_ill_* is the probability of illness, and *P_ill/in_* the probability of illness per infection. *P_ill/in_* values were obtained from Ahmed [21]. The risks of infection per day was calculated using values for the months of August, September, and October 2018, while the risks of infection per year were calculated based on average values for the three-month period.

Correlation analysis based on Spearman’s correlation coefficient (*Rs*) (Equation (4)) was used to determine the relationship between selected water quality parameters at a significance level (α) of 0.05. Since water quality data are commonly not normally distributed, their correlation needs to be determined using non-parametric statistics, such as *Rs*. *Rs* performs the correlation analysis based on ranked data, rather than actual values [22]. It does not require any assumptions regarding the frequency distribution of the variables, and is not sensitive to outliers [23]. *Rs* has been used in correlation analyses in groundwater quality studies, including [23,24,25]. *Rs* was calculated in Equation (4) in Microsoft Excel (2016, Microsoft, Johannesburg, South Africa).

(4) $$Rs\;=\;\frac{\sum \left(x\;-\;\tilde{x}\right)\left(y\;-\;\tilde{y}\right)}{\sqrt{\sum {\left(x\;-\;\tilde{x}\right)}^{2}{\left(y\;-\;\tilde{y}\right)}^{2}}}$$

where *x* and *y* are the ranks of water quality variables, and $\tilde{x}$ and $\tilde{y}$ are mean ranks of *x* and *y*, respectively. The correlation was done for selected water quality variables to assist in determining the potential sources of groundwater contamination. The relationship between parameters was characterised as strong, moderate, and weak when correlation coefficient (R) values were in the ranges of +0.8 to +1.0 and −0.8 to −1.0, +0.5 to +0.8 and −0.5 to −0.8, and +0.0 to 0.5 and −0.0 to −0.5, respectively [26]. The selected variable was correlated with the corresponding variable for all the sites, resulting in 10 values used in correlation analysis for each set of variables. The correlation of water quality parameters was statistically significant if the calculated *p*-value was less than α (0.05).

## 3. Results and Discussion

### 3.1. Suitability of Groundwater

**Figure 2:**
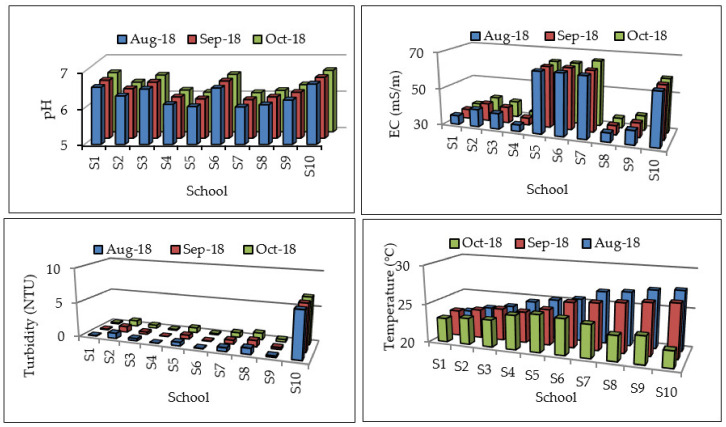
Physical parameters in groundwater samples.

**Figure 3:**
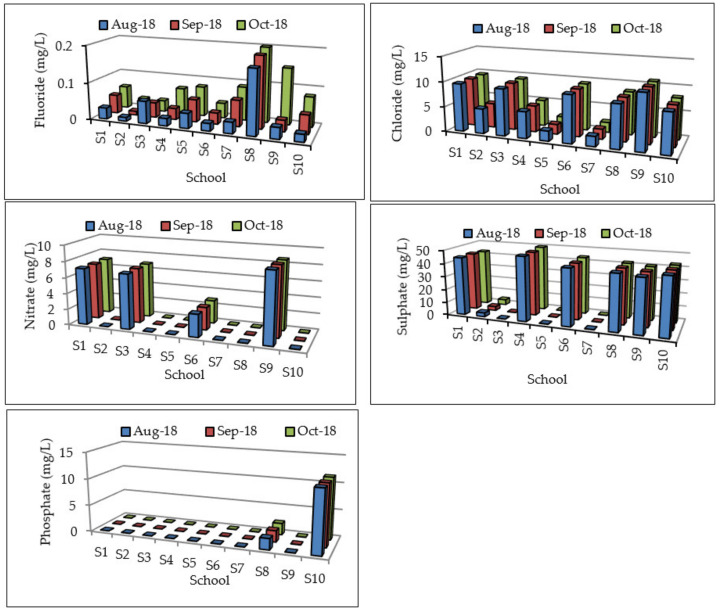
Fluoride, chloride, nitrate, sulphate, and phosphate concentrations (mg/L) in groundwater.

The pH and EC values for all sites were within DWAF [16] guidelines for domestic water use of 6–9 and 70 mS/m, respectively (Figure 2). S10 is the only school where turbidity values exceeded the recommended DWAF and WHO limits of 1 and 5 NTU, respectively, with its turbidity values ranging from 6.61–6.75 NTU. Turbidity exceeding 1 NTU increases chances of transmission of disease by micro-organisms. The temperature of groundwater varied from 22.1 to 27.6 °C (Figure 3). There is no specified guideline for temperature in water used for drinking and domestic purposes. However, high temperature negatively impacts on water quality by enhancing the growth of micro-organisms, which may increase taste, odour, colour, and corrosion problems [27].

**Figure 4:**
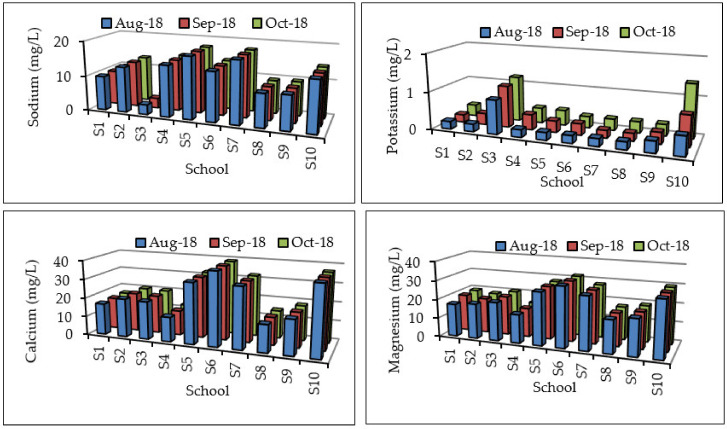
Sodium, calcium, potassium, and magnesium concentrations (mg/L) in groundwater samples.

Fluoride, chloride, sulphate, sodium, and potassium were within the recommended water quality guidelines for domestic water use of 1, 100, 200, 100, and 50 mg/L, respectively (Figure 3 and Figure 4), and hence had no potential health effects. Nitrate concentrations for S1, S3, and S9 exceeded the DWAF [16] guideline of 6 mg/L and are associated with rare instances of methaemoglobinaemia in infants and no effects in adults. The nitrate concentrations ranged from 7.03–7.08, 6.86–6.88, and 8.66–8.69 mg/L for S1, S3, and S10, respectively. Phosphate was not detected in most of the samples except for schools S8 and S10, with the concentrations ranging from 2.05 to 2.14 and 11.45 to 11.59 mg/L, respectively. DWAF [16] and WHO [17] have not specified guidelines for phosphate in drinking water. Phosphate in groundwater may be of concern, as it can provide a pathway for contamination of surface water bodies, resulting in eutrophication [28]. Odiyo et al. [29] could not detect phosphate in groundwater because its concentration is naturally low (usually less than a few tenths or hundredths of mg/L). Calcium and magnesium concentrations for the majority of the sites were within the recommended DWAF [16] water quality guidelines for domestic water use. Calcium concentrations in groundwater from schools S6 and S10 slightly exceeded the DWAF [16] guideline for domestic water use of 32 mg/L. Magnesium concentration in S6 slightly exceeded the guideline for domestic water use of 30 mg/L. Calcium and magnesium have no health effects, but are associated with increased scaling of appliances and reduced lathering of soap.

**Figure 5:**
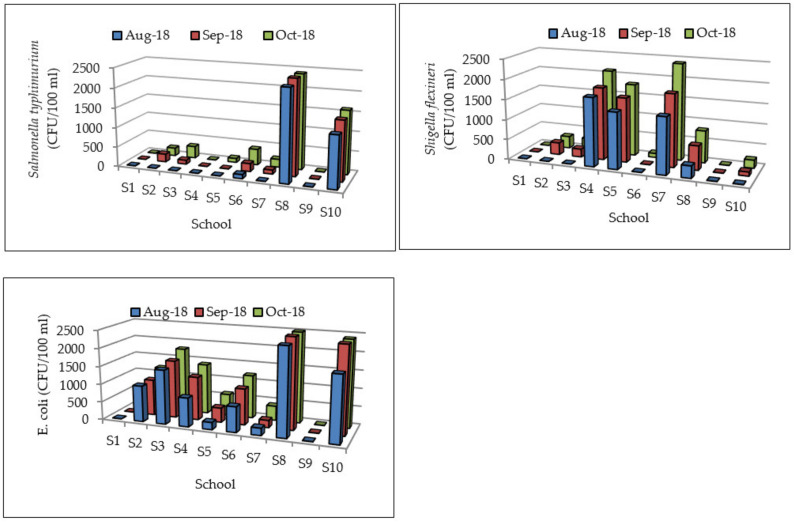
*Salmonella typhimurium*, *Shigella flexneri*, and *E. coli* in groundwater.

The acceptable levels of *Salmonella typhimurium* and *Shigella flexneri* in drinking water are not specified by the WHO [17,30]. The WHO [30] specified that *E. coli* should not be detectable in any 100 mL sample. SANS [18] noted that for water to be good and acceptable for drinking purposes, it should not have any trace of pathogens. Thus, counts of microbial organisms in drinking water should be zero. S1, S4, and S9 (for August 18 to October 18), S5 (for August 18 and September 18), and S2, S3, and S7 (for August 18) had no counts of *Salmonella typhimurium*, while S6, S8, and S10 had counts of *Salmonella typhimurium* for the 3 months studied (Figure 5). S1 and S9 (for August 18 to October 18), S2, S3, and S10 for August 18, S6 for August 18 and September 18 had no counts of *Shigella flexneri*, while groundwater in the rest of the schools (S4, S5, S7, and S8) had counts of *Shigella flexneri* for the 3 months studied. S1 and S9 had no counts of *E. coli* throughout the study period. *E. coli* was detected in groundwater in the rest of the schools (S2, S3, S4, S5, S6, S7, S8, and S10) in all the 3 months of study. There was no microbial contamination of groundwater in S1 and S9, since the boreholes are located far away from pit latrines. Groundwater from the majority of the schools therefore posed risks on the health of school children, and was not suitable for use due to microbial contamination. S1 and S9 with elevated nitrate concentrations had least potential health risks associated with rare instances of methaemoglobinaemia in infants and no effects in adults, and hence their groundwater was most suitable for use.

### 3.2. Microbial Risk Assessment

The results of water quality analysis showed that groundwater from a high proportion of schools (8 out of 10 had *E. coli* and *Shigella flexneri*, and 7 out of 10 had *Salmonella typhimurium*) had risks associated with pathogenic infections due to microbial contamination of groundwater. The microbes make the water hazardous and can cause infections and illnesses to school children. *E. coli*, *Salmonella typhimurium*, and *Shigella flexneri* are some of the commonly used reference/pathogens in QMRA. Health effects of these water quality parameters are provided in Table 2. Ingested doses of *E. coli* (0.00–2.50 CFU/day) in groundwater were generally higher than those of *Salmonella typhimurium* (0.00–2.37 CFU/day) and *Shigella* (0.00–1.87 CFU/day) (Table 3). The highest ingested doses for *E. coli* and *Salmonella typhimurium* were from S8, while S4 and S7 had the highest doses of *Shigella flexneri*. The ingested doses for *E. coli*, *Salmonella,* and *Shigella* obtained by [21] in northern and central Sindh districts in India where 64–94% of schools rely on groundwater ranged from 3.31–92.5, 19.8–189.6, and 35.2–480 CFU/day, respectively. The ingested doses obtained in this study are lower than those obtained by [21].

**Figure 6:**
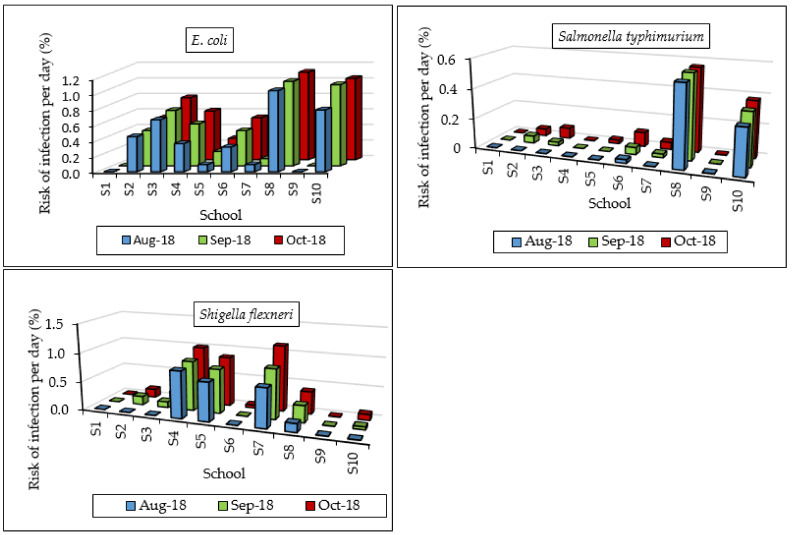
Risk of infection per day.

**Figure 7:**
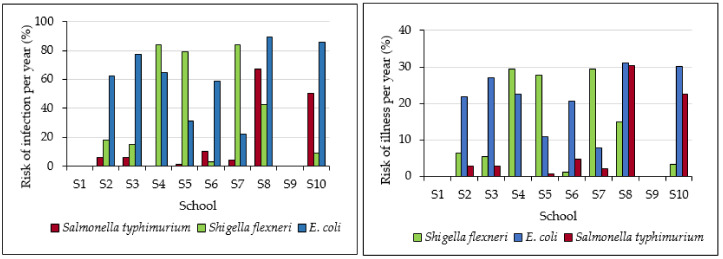
Risks of infection and illness per year.

Risks of infection per day were relatively low, with maximum values of 1.13, 0.56, and 1.13% for *E. coli*, *Salmonella typhimurium*, and *Shigella flexneri*, respectively (Figure 6). There was a relatively high annual risk of infections due to *E. coli* and *Shigella flexneri* for a majority of the schools (Figure 7) with highest values of 89.11 and 83.75%, respectively. Risk of annual infection due to *Salmonella typhimurium* was the lowest, with only two schools (S8 and S10) with values above 50%. Groundwater from S8 had the highest risk of annual infections of 89.11 and 67.50% due to *E. coli* and *Salmonella typhimurium*, respectively. Maximum risks of illness per year were 31.19, 30.37, and 29.31% for *E. coli*, *Salmonella typhimurium*, and *Shigella flexneri*, respectively. Ahmed et al. [21] reported annual risks of infection of 97.9–100%, 100%, and 80.9–100%, while risks of illness were 34.9–35, 45, and 28.3–35.0, for *E. coli*, *Salmonella typhimurium*, and *Shigella flexneri*, respectively. These were higher than annual risks of infections and illness obtained in this study. Machdar et al. [19] reported 99.7% risk of infection by *E. coli* from communal wells in Accra, Ghana, where children constituted 30% of the total population using groundwater.

*E. coli* had higher ingested doses, risks of infection, and illness per year compared to *Salmonella typhimurium* and *Shigella flexneri.* This indicates a high prevalence of diseases associated with *E. coli* in the study area. The results are comparable with those of a study by Taonameso et al. [14], which indicated that 25% of 125 boreholes sampled within the Vhembe District Municipality had a risk of causing infection based on *E. coli* counts > 10 per 100 ml. *E. coli*, *Salmonella typhimurium*, and *Shigella flexneri* are some of the prevalent diarrhoeagenic pathogens in groundwater used in rural areas of Limpopo Province [4,12]. Bessong et al. [10] indicated prevalence of diarrhoea in groundwater associated with *E. coli*, *Salmonella typhimurium*, and *Shigella* in the Vhembe District Municipality. This indicates that these pathogens are a major threat to the health of communities in Vhembe District, including school children. There is therefore a need to protect groundwater sources from microbial contamination. *E. coli*, *Salmonella typhimurium*, and *Shigella* are also some of the well-known causes of gastrointestinal diseases all over the world [33], constituting a major threat to public health. The study managed to pinpoint risks associated with groundwater consumption based on short-term data, as was similarly done in other published studies, such as Machdar et al. [19] and Ahmed et al. [21].

### 3.3. Correlation of Selected Water Quality Parameters

Results of correlation analysis (Table 4) indicated strong positive correlation of EC with calcium and magnesium from August 18 to October 18, with *Rs* values greater than 0.8. The correlations were not statistically significant, since *p*-values were greater than α (0.05). EC is directly related to the concentration of ionised substances in water, including calcium and magnesium; hence, they are positively correlated. Shrestha and Basnet [34] noted that calcium and magnesium strongly contributed to change in the EC values, with *Rs* values of 0.762 and 0.802, respectively. Positive correlations of EC with calcium and magnesium also indicate that these ions dominate groundwater in a region [35] and are likely to be derived from the same sources, such as chemical fertilisers, domestic discharges, and industrial effluents [36]. In groundwater within metamorphic rocks, such as in the study area, calcium naturally occurs at low concentration, while magnesium concentrations are low in groundwater due to its generally lower abundance [37]. Thus, elevated concentrations of calcium and magnesium may be due to anthropogenic sources. In the study area, the sources of calcium and magnesium are likely to be chemical fertilisers and domestic waste.

The correlations of sodium with EC and *Shigella flexneri*, and phosphate with *Salmonella typhimurium* were moderate and positive (Table 2). However, their correlations were not statistically significant, except for phosphate with *Salmonella typhimurium* from September 18 to October 18. A positive correlation of EC and sodium, similarly to that of calcium and magnesium, indicates that sodium also dominates groundwater, as noted by Kumar et al. [35], and is derived from similar sources. Phosphate is moderately correlated to *Salmonella typhimurium* because they may be from similar sources of pollution. Low phosphorus concentrations in pristine groundwater are often increased due to anthropogenic sources, such as fertilizers, manure, or sewage [38]. *Salmonella typhimurium* and *Shigella flexneri* also originate from faecal contamination of groundwater by human (from sewage or pit latrines) or animal waste (manure).

Nitrate and chloride had a positive and strong correlation, which was not statistically significant. Correlation of nitrate and chloride is attributed to anthropogenic sources, including fertilizers, sewerage, animal waste, organic manure, and pit latrines [39]. Odiyo and Makungo [40] attributed a high correlation of nitrate and chloride to faecal contamination of groundwater in Siloam Village, South Africa. Kohn et al. [41] indicated that a correlation between nitrates and chlorides suggests that they may be potentially derived from the same source (manure from agricultural activities). The schools in the study area use pit latrines for sanitation, which are potential sources of faecal contamination. This suggests that a positive correlation of nitrates and chlorides in groundwater from the schools is linked to anthropogenic sources from agricultural activities and faecal contamination from pit latrines. The correlations of sodium with chloride were moderate, negative and statistically significant (*p*-values less than 0.05). Sodium and chloride ions enter the solution in equal quantities during the dissolution of halite (sodium chloride), resulting in a linear positive relationship which is close to 1 [42]. Negative correlation between sodium and chloride would therefore indicate that there are other processes which release sodium into the groundwater. Sajil Kumar and James [43] also indicated a positive correlation between sodium and chloride would exist if they originated from a common source. This supports the suggestion that the negative correlation between sodium and chloride obtained in this study indicates that they are from different sources.

The *p*-values for most of the water quality parameters were greater than 0.05, indicating that the correlations were mostly not significant. This is supported by the results of water quality analysis, where the majority of chemical water quality parameters were within recommended guidelines for domestic use. However, the results of this study are still important to inform measures for preventing chemical contamination of groundwater, which may increase the health risks to school children. Groundwater is the sole source of water supply to the schools, and it is therefore crucial to safeguard its quality.

The results of the correlation analysis indicate that the potential sources of groundwater contamination include chemical fertilisers, domestic waste, and faecal contamination from agricultural activities, residential areas and pit latrines, respectively. The schools are in an area where there are school gardens, cultivated lands and residential settlements where these contaminants are generated. Microbial contamination of groundwater is attributed to pit latrines, as the results of the study indicated high levels of pathogens in schools where boreholes were located within the proximity of pit latrines (4–7 m). Residential settlements and cultivated lands also cover significant portions (41 and 48%, respectively) of the study area, and hence contribute to groundwater contamination.

## 4. Conclusions

Most of the water quality parameters were within the recommended guidelines for domestic water use. Calcium, magnesium, and nitrates slightly exceeded the guidelines in some of the groundwater samples. A major threat to the health of school children was microbial contamination of groundwater. The results of the study indicated that groundwater from only 2 out of the 10 schools had no counts of *E. coli*, *Shigella flexneri*, and *Salmonella typhimurium*. The schools S1 and S9 had most suitable groundwater for school children with the only potential health risk being rare instances of methaemoglobinaemia in infants, due to slightly high nitrate concentrations. Groundwater from the rest of the schools had major potential health risks due to poor microbial quality. High levels of microbial microorganisms in groundwater from most of the schools and elevated concentrations of calcium, magnesium, phosphate, and nitrates in some of the schools provided evidence that there are pathways for contamination of groundwater through the geological formation. The QMRA indicated that though the risk of infection per day was relatively low, there was a relatively high annual risk of infections due to *E. coli* and *Shigella flexineri* for a majority of the schools with highest values of 89.11 and 83.75%, respectively. *E. coli* had higher ingested doses, risks of infection, and illness per year compared to *Salmonella typhimurium* and *Shigella flexneri.*

The results of the Spearman correlation analysis indicated potential contamination of groundwater by anthropogenic sources, such as agricultural activities and faecal contamination from pit latrines. Though most of the correlations were not statistically significant, the findings are still critical to inform decision-making. Groundwater was the sole source of water supply to the schools due to a lack of piped water supply. Preventive measures to minimise the risks of groundwater contamination and use of contaminated groundwater are therefore essential. A study by Odiyo and Makungo [40] found out that the nearer the pit latrine, the higher the risk of faecal contamination. Pit latrines should therefore be located far from boreholes (the recommended minimum safe distance by Xu and Braune [44] is more than 50 m) to minimise microbial contamination of groundwater. Application of agricultural fertilisers should be limited or controlled to minimise groundwater pollution. It is also essential to implement point-of-use or abstraction treatment of groundwater by the schools. There is a need to discuss the strategies with school officials to promote their implementation and prevention/minimisation of potential health risks. Though this study was done based on limited data, it managed to pinpoint risks associated with groundwater consumption based on short-term data, as was similarly done in other published studies, such as Machdar et al. [19] and Ahmed et al. [21]. Since the study was limited to a three-month sampling period (August to October 2018), it is important to undertake further studies based on medium- to long-term data which will clearly capture seasonal variations of water quality and associated health risks. These findings will provide background data and information for future studies.

## Figures and Tables

**Table 1:** Dose-response assessment parameters.

Pathogen	Parameters Used in Estimation
*E. coli*	α = 0.084; β = 1.44 (for children); *P_ill/in_* = 0.35
*Salmonella typhimurium*	α = 0.33; β = 1.39; *P_ill/in_* = 0.45
*Shigella flexineri*	α = 0.41; β = 42.86; *P_ill/in_* = 0.35

**Table 2:** Health effects of pathogens in groundwater.

Pathogen	Schools	Health Effects
*Salmonella typhimurium*	S2, S3, S5, S6, S7, S8 and S10	Gastroenteritis (ranging from mild to fulminant diarrhoea, nausea and vomiting), bacteraemia or septicaemia (high spiking fever with positive blood cultures), and a carrier state in persons with previous infections ^1^
*Shigella flexneri*	S2, S3, S4, S5, S6, S7, S8 and S10	Formation of ulcers, blood-stained diarrhoea and elevated levels of neutrophils in faeces ^2^
*E. coli*	S2, S3, S4, S5, S6, S7, S8 and S10	Nausea, fever, vomiting, headaches, abdominal cramps and chills ^2^

^1^ WHO [31] and ^2^ WHO [32].

**Table 3:** Average ingested dose of bacteria (CFU/day).

School	*E. coli*	*Salmonella typhimurium*	*Shigella flexneri*
S1	0.00	0.00	0.00
S2	1.07	0.13	0.20
S3	1.63	0.13	0.17
S4	1.13	0.00	1.87
S5	0.40	0.03	1.60
S6	0.97	0.23	0.03
S7	0.27	0.10	1.87
S8	2.50	2.37	0.57
S9	0.00	0.00	0.00
S10	2.20	1.47	0.10

**Table 4:** Results of correlation analysis.

Parameters	Aug-18		Sep-18		Oct-18	
	*Rs*	*p*-Value	*Rs*	*p*-Value	*Rs*	*p*-Value
EC and calcium	0.965	0.3800	0.9391	0.670	0.954	0.563
EC and magnesium	0.879	0.364	0.854	0.503	0.782	0.406
Sodium and EC	0.462	0.119	0.527	0.200	0.539	0.207
Sodium and *Shigella flexneri*	0.712	0.956	0.577	0.244	0.518	0.208
Phosphate and *Salmonella typhimurium*	0.796	0.0691	0.648	0.002	0.651	0.0016
Nitrate and chloride	0.837	0.4579	0.861	0.360	0.837	0.457
Sodium and chloride	−0.730	**0.0001**	−0.770	**0.0001**	−0.721	**0.0003**

Values in bold indicate a statistically significant correlation.
